# Direct conversion of theophylline to 3-methylxanthine by metabolically engineered *E. coli*

**DOI:** 10.1186/s12934-015-0395-1

**Published:** 2015-12-21

**Authors:** Khalid H. R. Algharrawi, Ryan M. Summers, Sridhar Gopishetty, Mani Subramanian

**Affiliations:** Department of Chemical and Biochemical Engineering, The University of Iowa, Coralville, IA 52241 USA; Department of Chemical and Biological Engineering, The University of Alabama, Tuscaloosa, AL 35487 USA; Center for Biocatalysis and Bioprocessing, University of Iowa Research Park, The University of Iowa, 2501 Crosspark Road-Suite C100, Coralville, IA 52241 USA; Department of Chemical Engineering, University of Baghdad, Baghdad, Iraq

**Keywords:** 3-methylxanthine, 1-methylxanthine, Theophylline, *E. coli*, Biocatalyst, *N*-demethylation, Preparative chromatography, Metabolic engineering

## Abstract

**Background:**

Methylxanthines are natural and synthetic compounds found in many foods, drinks, pharmaceuticals, and cosmetics. Aside from caffeine, production of many methylxanthines is currently performed by chemical synthesis. This process utilizes many chemicals, multiple reactions, and different reaction conditions, making it complicated, environmentally dissatisfactory, and expensive, especially for monomethylxanthines and paraxanthine. A microbial platform could provide an economical, environmentally friendly approach to produce these chemicals in large quantities. The recently discovered genes in our laboratory from *Pseudomonasputida*, *ndmA, ndmB*, *and ndmD*, provide an excellent starting point for precisely engineering *Escherichia coli* with various gene combinations to produce specific high-value paraxanthine and 1-, 3-, and 7-methylxanthines from any of the economical feedstocks including caffeine, theobromine or theophylline. Here, we show the first example of direct conversion of theophylline to 3-methylxanthine by a metabolically engineered strain of *E. coli*.

**Results:**

Here we report the construction of *E. coli* strains with *ndmA* and *ndmD*, capable of producing 3-methylxanthine from exogenously fed theophylline. The strains were engineered with various dosages of the *ndmA* and *ndmD* genes, screened, and the best strain was selected for large-scale conversion of theophylline to 3-methylxanthine. Strain pDdA grown in super broth was the most efficient strain; 15 mg/mL cells produced 135 mg/L (0.81 mM) 3-methylxanthine from 1 mM theophylline. An additional 21.6 mg/L (0.13 mM) 1-methylxanthine were also produced, attributed to slight activity of NdmA at the *N*_*3*_-position of theophylline. The 1- and 3-methylxanthine products were separated by preparative chromatography with less than 5 % loss during purification and were identical to commercially available standards. Purity of the isolated 3-methylxanthine was comparable to a commercially available standard, with no contaminant peaks as observed by liquid chromatography-mass spectrophotometry or nuclear magnetic resonance.

**Conclusions:**

We were able to biologically produce and separate 100 mg of highly pure 3-methylxanthine from theophylline (1,3-dimethylxanthine). The N-demethylation reaction was catalyzed by *E. coli* engineered with N-demethylase genes, *ndmA* and *ndmD*. This microbial conversion represents a first step to develop a new biological platform for the production of methylxanthines from economical feedstocks such as caffeine, theobromine, and theophylline.

**Electronic supplementary material:**

The online version of this article (doi:10.1186/s12934-015-0395-1) contains supplementary material, which is available to authorized users.

## Background

Xanthine is a ubiquitous naturally occurring purine base. Other common naturally occurring xanthine derivatives include caffeine (1,3,7-trimethylxanthine), theobromine (3,7-dimethylxanthine), and theophylline (1,3-dimethylxanthine, TP), which are found in many foods, drinks, and pharmaceuticals [1–3]. Paraxanthine (1,7-dimethylxanthine) and 1-, 3-, and 7-methylxanthines are also naturally occurring xanthine derivatives, but are transient metabolites found at very low levels [1, 2, 4]. Several xanthine derivatives have also been synthesized chemically for use in the medical industry [5]. These compounds, natural and synthetic, have been shown to have various biomedical effects, with targets including adenosine receptors [6, 7], phosphodiesterases [8, 9], calcium release channels [10–13], and GABA_A_ receptors [13, 14].

For example, 3-methylxanthine (3MX) has been assessed as an adenosine antagonist [6] and produces the same maximal relaxation of guinea pig tracheal muscle as does TP [15]. Given that 3MX is a metabolite of TP in humans [16], TP itself may be a prodrug; thus there is an interest in directly testing 3MX as well. 3MX and TP are also used to examine conformational heterogeneity in RNA aptamers and riboswitches [17, 18]. 1-methylxanthine (1MX) is an essential human urinary metabolite of caffeine and TP [19–22] and exhibits similar activities to other naturally occurring methylxanthines. Unlike caffeine, TP, and theobromine, 3MX and 1MX do not occur naturally at high levels in plants. Instead, 3MX and 1MX are currently produced only by chemical methods, which are difficult due to the challenge of achieving selective alkylation of each of the nitrogen atoms [23–26].

**Fig. 1:**
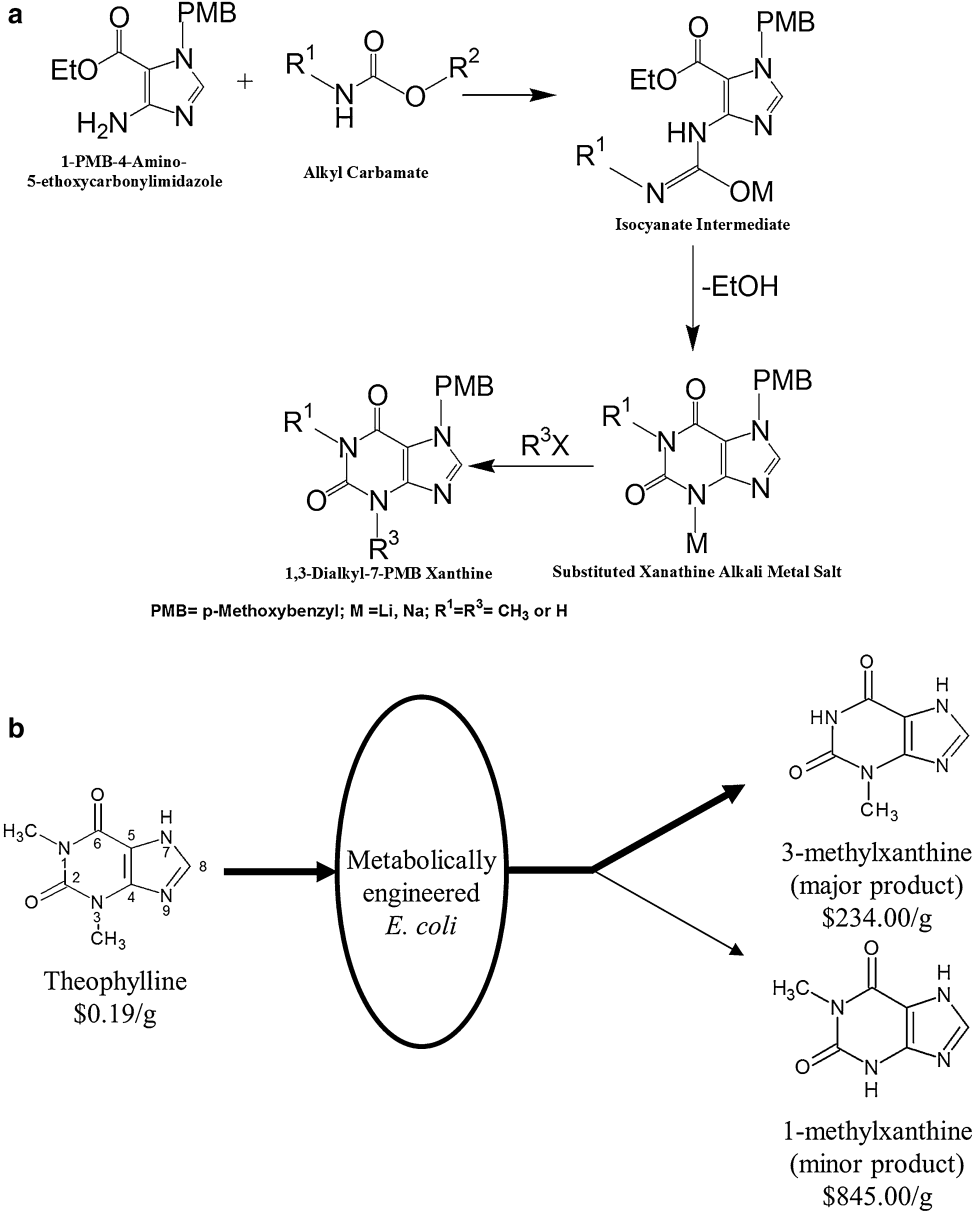
Methylxanthine production schemes. **a** Example of generalized synthetic methods adapted from [30]. **b** Production of 3MX (major product) and 1MX (minor product) from TP by metabolically engineered *E. coli* containing the *ndmA* and *ndmD* genes. Prices per gram given below the compounds are based on the Sigma Aldrich Catalog for the highest lot available (Additional file 1: Table S1)

Many purine alkaloids are traditionally produced via Traube synthesis, which uses the cyclization of 4,5-diaminopyrimidines with formic acid, dithioformic acid, or other carboxylic acids [27, 28]. Imidazoles are also used for the production of purines [29]. Zavialov et al. developed a practical method describing the synthesis of 1- and 1,3- substituted xanthines by reacting an imidazole precursor with carbamates in the presence of suitable base [30]. The reaction was carried out under inert conditions using solvents such as tetrahydrofuran, bis(2-methoxyethyl) ether, and toluene. About seven steps of synthesis were needed to get the targeted methylxanthine. Allwood et al. developed a highly efficient synthesis route to *N*-functionalized derivatives of xanthine by orthogonal safety-catch protection strategy using cyclocondensation of aminoimidazole with methyl-2-phenylthioethyl carbamates [31]. Liu et al*. s*ynthesized novel substituted xanthines by 46 routes [32]. In one of the routes, xanthine analogs containing substituents at the *N*_1_, *N*_3_, and *N*_7_ atoms were produced by treating 1,3-dialkyl-5,6-diaminouracils with triethylorthoformate. Traditionally, synthetic methods for the production of purine alkaloids utilize many undesirable chemicals, solvents, and harsh reaction conditions, and result in multiple reactions with undesired products; therefore, it is complicated and expensive (Fig. 1a). For synthesis of specific alkylxanthines, such as 3MX, additional deprotection steps are needed and the overall yield from starting material such as an imidazole precursor is highly variable, i.e., 65–90 %. However, the exact yield of each specific methylxanthine is not clear. At each step of the reaction, the intermediate needs to be purified before the next step. Three different solvents reportedly give different yields [30].

Caffeine and related methylxanthines are toxic to most bacteria and invertebrates [33, 34]. However, some bacteria, most of which are *Pseudomonads*, have evolved the ability to metabolize caffeine [35–42]. The bacterial strain *Pseudomonas putida* CBB5 degrades caffeine via sequential *N*-demethylation to theobromine (3,7-dimethylxanthine), 7-methylxanthine, and ultimately xanthine [38]. CBB5 can also *N*-demethylate TP to 1MX and 3MX, which are further *N*-demethylated to xanthine. This is the first bacterial strain reported to grow on and metabolize TP [38]. These same pathways for caffeine and TP metabolism have also been characterized genetically in *Pseudomonas* sp. CES [39].

The enzyme NdmA catalyzes the *N*_1_-demethylation of TP to 3MX. In addition, NdmA also converts caffeine to theobromine [43]. This enzyme is a Rieske (2Fe-2S) non-heme iron monooxygenase that requires a partner reductase, NdmD, to transfer electrons from NADH. The reaction requires one molecule of O_2_ per methyl group removed, resulting in the production of formaldehyde and water. We previously showed that the *ndmA* and *ndmD* genes are expressed partly in soluble form in *Escherichia coli* [43], and that a strain expressing both genes can be used to convert caffeine to theobromine [44].

Our broader interest is to generate a new, common platform for biocatalytic production of several high value methylxanthines via metabolically engineered *E. coli* (Fig. 1b) from cheaper feedstocks such as caffeine, TP and theobromine (see Additional file 1: Table S1 for relative value of each compound). There is a high-value differential between TP and desired product, 3MX (Fig. 1b). Our initial focus has been to produce 3MX from TP using *E. coli* engineered with *ndmA* and *ndmD.* Biocatalytically-produced 3MX, besides reagent market as well as potential pharmaceutical effects [6], has commercial application as a nutraceutical (unpublished, personal communication between senior author and two different nutraceutical companies). There are several suppliers of synthetic 3MX as reagents worldwide [45], but no current suppliers of 3MX produced through biocatalytic means. The preferred substrate of the NdmA enzyme is TP, with a k_cat_/K_M_ ratio for TP nearly double that of caffeine [43]. The present work is the first report on the biocatalytic production of 3MX. The genes *ndmA* and *ndmD* were introduced into *E. coli* at different gene dosages, and the resultant strains were screened for 3MX production. The optimum strain with the highest 3MX production was chosen for further study, including small-scale production of 3MX to dispatch to clients. NdmA produced 1MX as a minor product as a result of non-specific N-demethylation at the *N*_3_-position. 1MX was not fully characterized since this is not the best method to produce this fine chemical. The biocatalytic approach used here operates at ambient temperature and pressure and is environmentally friendly. In contrast, chemical synthesis of methylxanthines uses several chemicals, multiple reactions, and non-ambient reaction conditions (Fig. 1a).

## Results and discussion

### Initial screening of growth and 3MX production by metabolically engineered *E. coli*

**Fig. 2:**
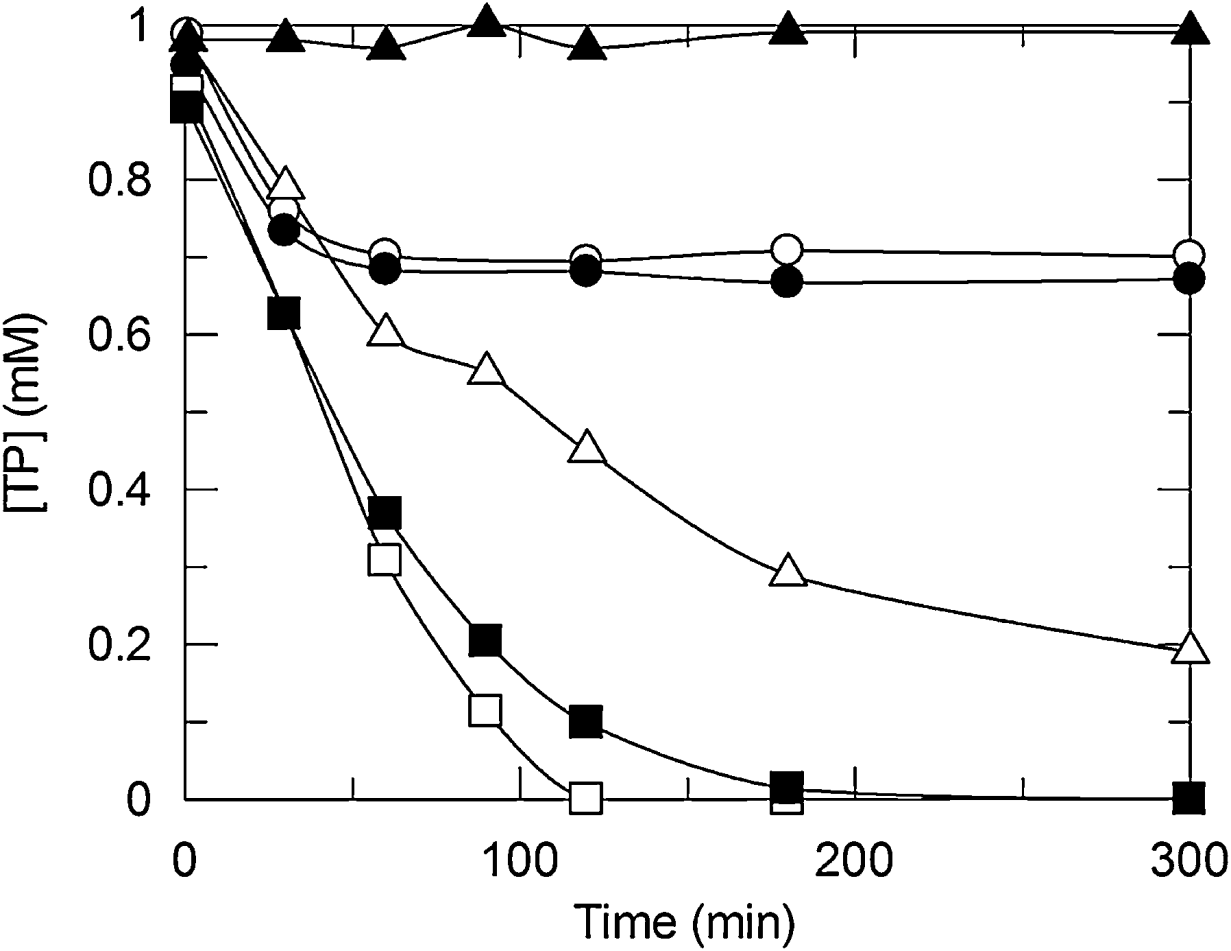
Degradation of TP by metabolically engineered *E. coli* resting cells. *Shaded triangle* strain BL21(DE3) (negative control); *Open circle* strain pAD1; *Shaded circle* strain pAD1dAA; *Open triangle* strain dDA; *Shaded square* strain pAD1dDA; *Open square* strain pAD1dDD. Cells (OD_600_ = 2.5) were incubated with 1 mM TP in 50 mM KP_i_ buffer at 30 °C with 400 rpm shaking, and metabolites were quantified via HPLC

All plasmids and strains used in this work are listed in Table 1, and plasmid maps are provided in Additional file 1: Figure S1. We first tested conversion of TP to 3MX using a strain of *E. coli* that contained plasmid pAD1 [23]. Resting cells (OD_600_ = 2.5) converted approximately 0.3 mM TP to 3MX over 1 h, after which the reaction essentially stopped (Fig. 2). In order to increase activity, plasmids dAA, dDD, and dDA were added to the strain carrying pAD1, resulting in three new strains. These new strains allowed us to test the effect of different levels of *ndmA* and *ndmD* copy numbers on 3MX production (see Additional file 1: Table S2 for approximate gene copy numbers of each strain). Addition of *ndmA* only (strain pAD1dAA) had very little effect on activity (Fig. 2). Increasing the copy number of both genes (strain pAD1dDA) greatly increased the activity over strain pAD1dAA, with nearly complete conversion in 3 h. However, increasing the *ndmD* gene copy number only (strain pAD1dDD) resulted in complete conversion of TP within 2 h (Fig. 2). Strain pAD1dDD, which contained the lowest *ndmA* copy number, exhibited a slightly higher activity than did strain pAD1dDA, suggesting that plasmid pAD1 provided a sufficient *ndmA* gene dosage. These results also indicated that the reaction was limited by the amount of soluble NdmD produced inside the cells, since the activity increased with increasing *ndmD* copy number.

**Table 1:** Plasmids and strains used in this study

Name	Characteristics	Source
Plasmids		
pAD1	Amp^R^, T7 promoter, *ndmA*, *ndmD*, rbsAD1, F1 origin	[44]
pET28-His-ndmD	Kan^R^, T7 promoter, *ndmD*, F1 origin	[43]
pACYCDuet-1	Expression vector, Cm^R^, 2 T7 promoters, p15A origin	Novagen
dA	pACYCDuet-1 with one copy of *ndmA*	This study
dA0	pACYCDuet-1 with one copy of *ndmA* and a second MCS	This study
dAA	pACYCDuet-1 with two copies of *ndmA*	This study
dD0	pACYCDuet-1 with one copy of *ndmD* and a second MCS	This study
dDD	pACYCDuet-1 with two copies of *ndmD*	This study
dDA	pACYCDuet-1 with one copy of *ndmD* and on copy of *ndmA*	This study
*E. coli strains*		
* E. coli* BL21(DE3)	F^−^ *ompT hsdS*_B_ (r_B_^−^m_B_^−^) *gal dcm* (DE3)	Invitrogen
* E. coli* pAD1^a^	BL21(DE3) pAD1	[44]
* E. coli* pAD1dDD	BL21(DE3) pAD1 dDD	This study
* E. coli* pAD1dDA	BL21(DE3) pAD1 dDA	This study
* E. coli* pAD1dAA	BL21(DE3) pAD1 dAA	This study
* E. coli* dDA	BL21(DE3) dDA	This study
* E. coli* pHisD	BL21(DE3) pET28-His-ndmD	[43]
* E. coli* pDdAA	BL21(DE3) pET28-His-ndmD dAA	This study
* E. coli* pDdA	BL21(DE3) pET28-His-ndmD dA	This study

^a^Strain pAD1 was originally named *E. coli* strain RMS1 in a previous publication [44]

In the case of plasmid pAD1, the *ndmD* gene is separated from the T7 promoter by approximately 1.1 kb of sequence containing the *ndmA* ribosomal binding site and gene, followed by a short synthetic ribosomal binding site of unknown strength just before the *ndmD* gene (Additional file 1: Figure S1). SDS-PAGE of strain pAD1 (Additional file 1: Figure S2) showed a strong band of soluble NdmA, but very little NdmD (soluble or insoluble). In contrast, strain pAD1dDD had very strong soluble and insoluble NdmD bands. Based on activity and electrophoretic analysis, plasmid pAD1 clearly did not produce sufficient soluble intracellular NdmD. This was confirmed using resting cells (OD_600_ = 2.5) of an *E. coli* strain containing only plasmid dDA, which consumed 0.8 mM TP over 300 min (Fig. 2). Plasmid dDA is based on the pACYCDuet-1 backbone, giving a plasmid (and gene) copy number approximately fourfold lower than that of pAD1. In spite of the lower overall gene dosage, activity was much higher in strain dDA than in strains pAD1 and pAD1dAA. Methods to increase expression of *ndmD* from plasmid pAD1 only could involve using a known strong ribosomal binding site and/or a second T7 promoter between *ndmA* and *ndmD*.

In order to increase intracellular levels of NdmD, a plasmid containing the *ndmD* gene placed immediately downstream of the T7 promoter and ribosomal binding site in pET28a(+) [43] was used. Compatible plasmids containing one or two copies of *ndmA* (plasmids dA and dAA, respectively) were then added to a strain of *E. coli* harboring pET28-His-ndmD. This resulted in strains with a low (pDdA) or medium (pDdAA) *ndmA* gene dosage, based on estimated copy number and number of genes in each plasmid. The activity and protein expression levels of these two strains were then compared with strain pAD1dDD, which had the highest *ndmA* dosage of the three (Additional file 1: Table S2). Strains pDdA, pDdAA, and pAD1dDD grew to a similar OD_600_ in 100 mL Luria–Bertani broth (LB) (Additional file 1: Table S3) when gene expression was induced as described in the “Methods” section. SDS-PAGE revealed that soluble (active) protein expression is about the same for NdmA and NdmD among the three strains (Additional file 1: Figure S2). Each wet cell paste was used to test the conversion of TP to 3MX by resuspending in KP_i_ buffer to a final cell concentration of 30 mg/mL and initial TP concentration of 4 mM. After 90 min of the reaction time, TP was reduced 56, 51, and 43 % by suspensions of pDdA, pDdAA, and pAD1dDD, respectively. Approximately 84, 82, and 81 % of the consumed TP was converted to 3MX in strains pDdA, pDdAA, and pAD1dDD, respectively, with the remaining TP forming 1MX (Additional file 1: Table S3). Based on these results, strain pDdA was chosen for further studies due to the highest yield of 3MX from TP. Clearly, the additional gene dosage of *ndmA* (pDdAA) did not improve 3MX yield, relative to single gene dose (pDdA). Therefore, the activity of the cells was proven to be independent of the *ndmA* gene dosage and highly dependent on the *ndmD* gene dosage and expression in each *E. coli* strain.

### Comparison of growth media

**Fig. 3:**
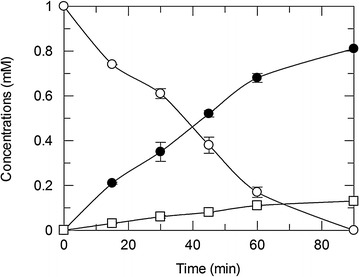
Production of methylxanthines from TP by strain pDdA grown in SB. 1 mM TP (*open circle*) was converted to 0.81 ± 0.002 mM 3MX (*shaded circle*) and 0.13 ± 0.002 1MX (*open square*) within 90 min by 15 mg/mL of resting cells. Concentrations reported are means with standard deviations of triplicate results

The effect of culture medium on cell growth and activity was also measured by growing strain pDdA in Luria–Bertani Lennox (LB) and super broth (SB) media. SB produced approximately 50 % more cells than did LB (Additional file 1: Table S4). Cells were resuspended to 15 mg/mL, and the initial TP concentration in activity assays was lowered to 1 mM in order to achieve complete conversion, which would facilitate downstream purification and product recovery. TP was completely consumed in SB-grown cells within 90 min (Fig. 3). After 2 h, nearly all of the TP was consumed in both reactions (Additional file 1: Table S4). 3MX yield from TP was 82–83 %, with an additional 12–13 % being 1MX. Because the cells are capable of performing both *N*_1_- and *N*_3_-demethylations on both TP and also 1- and 3MX, some small amount of xanthine was also formed from the monomethylxanthine products. These results demonstrate that the media composition had no significant effect on product ratio. Given the complete conversion of TP achieved in shorter time and 50 % more biocatalyst harvested from SB, this medium was chosen for the production of 3MX to supply clients.

Although yield of 3MX is high, minor production of 1MX decreases the overall yield of 3MX. The slight *N*_3_-demethylation of TP by NdmA to form 1MX is surprising and in contrast with our previous findings that NdmA is highly specific for the *N*_1_ methyl group of caffeine and TP [43]. We therefore tested the activity of strain pDdA on caffeine and observed a slight (<2 %) *N*_3_-demethylation activity to form paraxanthine (1,7-dimethylxanthine, data not shown). The enzyme in the previously reported work was expressed in *E. coli* BL21(DE3) with a C-terminal hexahistidine (His_6_) tag for facile purification and assayed in vitro, and produced only 3MX from TP. 1MX was shown to be produced from TP by the highly-specific *N*_3_-demethylase NdmB-His. The present study utilizes NdmA expressed in the same microbial chassis without the His_6_ tag, and the reaction is carried out in vivo. It is unclear whether performing the reaction in vivo, elimination of the His_6_ tag from NdmA, enzyme expression level, and/or enzyme solubility [46] are involved in the change in site specificity. In our in vitro studies, the minimum amount of enzyme was used in order to determine the kinetics [43], and the paraxanthine and 1MX products may have been below the detection limit. However, the reduction in enzyme expression level (comparing strains pAD1 and dDA vs. strain pDdA) in this work did not result in a lower ration of products. Clearly, an in vitro approach would not be economical, as it would require addition of external NADH. It should be noted, however, that addition of a His_6_ tag has been implicated in changing substrate specificity of the thioesterase I in *E. coli* due to a slight change in active site geometry [47]. The reason for the discrepancy between NdmA and NdmA-His_6_ is currently under investigation. The original strain of *P. putida* CBB5 produced approximately twice as much 3MX as 1MX [38], however, the 1MX production in this strain, besides slight specificity of NdmA at *N*_3_-position, can mostly be attributed to NdmB [43]. Future work to reduce the *N*_3_-demethylation activity of NdmA in vivo when expressed in *E. coli* should create a more efficient process for production of 3MX, while simultaneously simplifying downstream recovery of 3MX.

### Larger scale reaction, preparative chromatography, and purification of 3MX

**Fig. 4:**
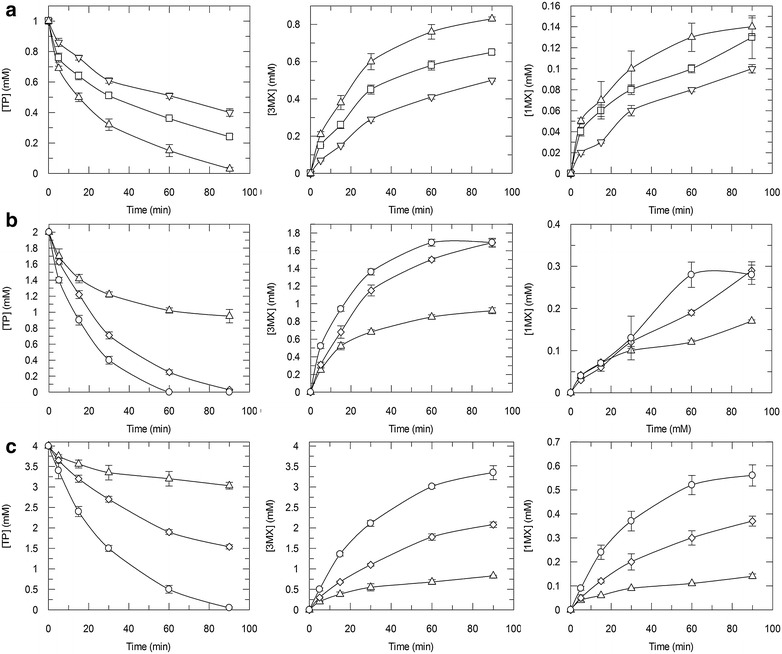
Effect of cell and substrate concentrations on 3MX production by *E. coli* pDdA. Resting cell assays were performed using 5 (*open triangle*), 10 (*open square*), 15 (*open triangle*), 30 (*open diamond*), and 60 (*open circle*) mg/mL wet cells. TP concentrations were 1 mM (**a**), 2 mM (**b**), and 4 mM (**c**). Concentrations of TP (*left*), 3MX (*middle*), and 1MX (*right*) are shown as means with standard deviations of triplicate reactions

The reaction conditions for conversion of TP to 3MX were optimized by evaluating different concentrations of cells (5, 10, 15, 30, and 60 mg wet cells/mL) and initial substrate concentration (1, 2, and 4 mM TP). It is clear from the data presented in Fig. 4 that a reaction containing 1 mM TP and 15 mg/mL resting cells provides linear conversion of TP to 3MX. At these reaction conditions, the product concentration and reaction volume suited the prep high pressure liquid chromatography (HPLC) column for complete product recovery.

Production of 3MX was scaled up by growing the pDdA strain in SB media in four 2.8 L Fernbach flasks, resulting in a total yield of 20 g wet cells. The cell yield was sufficient to perform a 1.3 L reaction with an initial TP concentration of 1 mM at 15 mg/mL resting cell suspension. Initial analysis by HPLC showed complete consumption of TP after 2 h of reaction time with formation of 0.81 and 0.13 mM 3MX and 1MX, respectively. The products were separated by preparative chromatography (Additional file 1: Figure S3). Resolution of 3MX (retention time of 116 min) and 1MX (retention time of 136 min) was sufficient to collect each of the two products separately. The two products were crystallized through evaporation and freeze-drying, resulting in 106 mg 3MX and a minor amount of 1MX. Because the very small amount of 1MX produced could not be collected from the walls of the freeze dryer tray, 1MX was not further characterized. We are attempting to produce 1MX from TP via a metabolically engineered *E. coli* host containing *ndmB* and *ndmD*. The NdmB enzyme has been shown to be highly specific for *N*_3_-demethylation [43], and a purified NdmB-His_6_ produced only 1MX in vitro.

The theoretical amount of 3MX produced in the reaction was 175 mg (~81 % mole to mole conversion from TP); however 36 % of the post-reaction mixture was used to optimize the preparative chromatographic separation. Therefore, a total of 111 mg 3MX (64 % of the post-reaction mixture) was loaded onto the column for purification and recovery. The resulting 106 mg pure 3MX indicates very little loss during separation with a purification yield of 95.5 % after optimization of separation in the prep column. Improving the selectivity of NdmA so that it only produces 3MX from TP would further increase the yield.

The reaction conditions described here could produce 135 mg/L 3MX. To our knowledge, this is the first report describing the non-transient microbial production of 3MX. Until now, all microbial production of 3MX has been as an intermediate in the caffeine and TP catabolic pathways [38, 48]. Therefore, there are no values in the literature with which to compare this yield. However, there was adequate amount for further analytical work and supply of samples to our clients.

Because the *ndm* genes have only recently been discovered [43, 46], previous attempts to produce methylxanthines through a biocatalytic route have focused primarily on metabolism and enzymology studies for conversion of caffeine to theobromine. Research has shown that addition of certain divalent metal ions, such as Co^2+^, Ni^2+^, Cu^2+^, and Zn^2+^ have a strong inhibitory effect on degradation of theobromine accumulated from caffeine in whole cells of *P. putida* [49, 50]. However, there are no known specific inhibitors to stop the reaction at the desired, high-value methylxanthines such as paraxanthine and 1-, 3-, and 7-methylxanthine. Also, this approach would not be optimal for methylxanthine production, as the wild type *P. putida* strains (CBB5 and others) have lower growth rates and cannot produce the same amount of enzyme (hence, catalytic activity) as can *E. coli* expressing the recombinant *ndm* genes. Jin et al. [51] cloned genes from the caffeine biosynthetic pathway of coffee and tea into *Saccharomyces cerevisiae*. The resulting strain produced a very low level (0.38 mg/L) of caffeine when fed exogenous xanthosine. Without addition of xanthosine, no caffeine was detected. Besides the low production level, use of plant genes restricts the possible products to 7-methylxanthine, theobromine, and caffeine, which are the metabolites of the caffeine biosynthetic pathway. Caffeine is mostly produced during the decaffeination of coffee beans [52, 53]. Theobromine and TP are mostly produced synthetically [54, 55], although extraction from plants is possible [56]. Thus, further strain optimization and protein engineering will be required before use of plant-based genes can be used in a microbial system to produce high value methylxanthines.

### Analytical characterization of biologically produced 3MX

**Fig. 5:**
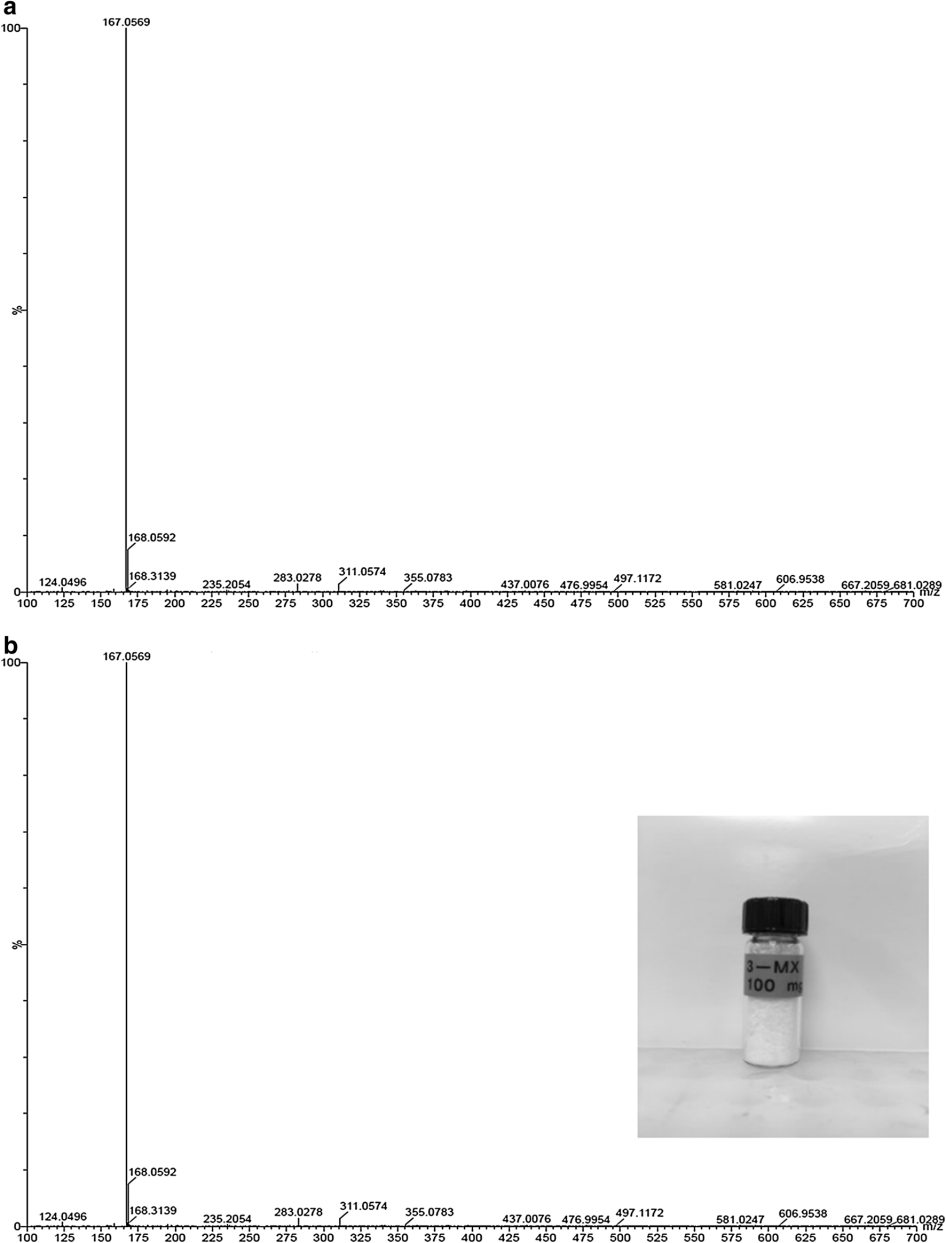
LC–MS spectra of 3MX samples. **a** Spectrum of 3MX standard purchased from Sigma–Aldrich. **b** 3MX produced in this work. Inset to **b**: Purified, crystallized 3MX produced in this work

The purity of both 3MX and 1MX was analyzed by analytical HPLC using appropriate authentic standards. The retention time of the biologically produced products (Additional file 1: Figure S3) and authentic standards were identical. The High Resolution LC-MS spectrum of biologically produced and standard 3MX matched very well (Fig. 5) and corresponded to the published spectrum [57]. LC/MS was recorded on ESI positive mode; distinct M + 1 ion peak at 167.0569 m/z was observed both in the standard (Fig. 5a) and the biologically produced 3MX (Fig. 5b).

The ^1^H NMR spectrum of biologically produced and standard 3-methyl xanthine also matched very well (Additional file 1: Figure S4). ^1^H NMR was recorded on a Bruker 500 MHz spectrophotomer using DMSO-d_6_ as solvent. Standard 3-methylxantine showed presence of peaks at *δ* 13.48 (s, 1H) and 11.07 (s, 1H) corresponding to –NH proton, and peaks at *δ* 8.01 and 3.3 corresponding to –C = H (s, 1H) and –CH_3_ group (s, 3H). The biologically produced 3MX also showed peaks at *δ* 13.48 (s, 1H) and 11.07 (s, 1H) corresponding to –NH proton, and peaks at *δ* 8.0 and 3.3 corresponding to –C = H (s, 1H) and –CH_3_ group (s, 3H).

## Conclusions

Our present work describes, for the first time, biocatalytic production of 3MX from TP with high yield. The process is carried out under ambient conditions in a single step using metabolically engineered *E. coli*. The larger vision of our work is to produce several high-value methylxanthines using specific combinations of *ndm* genes metabolically engineered in *E. coli* while choosing the best feedstock to get the highest yield of the specific product. This represents a new biocatalytic platform for production of methylxanthines using multiple cheap feedstocks and a common process of fermentation to yield biocatalyst, reaction conditions, and separation process.

## Methods

### Chemicals and reagents

TP, 1MX, 3MX, and xanthine were purchased from Sigma–Aldrich (St. Louis, MO). Luria–Bertani Lennox (LB) and Difco Select APS™ Super Broth (SB) dehydrated media were obtained from Becton–Dickinson and Company (Sparks, MD). Isopropyl β-D-thiogalactopyranoside (IPTG) was obtained from RPI Corp. (Mt. Prospect., IL). All PCR reactions were performed with Phusion HF polymerase from New England Biolabs (Ipswich, MA). Restriction enzymes and PCR reagents were also purchased from New England Biolabs. PCR primers were ordered from Integrated DNA Technologies (Coralville, IA). HPLC-grade methanol (J.T. Baker, Phillipsberg, NJ) was used in all chromatographic studies.

### Plasmid construction

All plasmids and strains used in this work are listed in Table 1. The pACYCDuet-1 vector backbone, which has a low-to-medium copy number of 10–12, was used for plasmids dAA, dA, dDD, and dDA. Plasmid dA was created by adding a single copy of *ndmA* in a manner that removed the second multiple cloning site. Plasmids pAD1 and pET28-His-ndmD contain the pET32a(+) and pET28a(+) vector backbones, respectively, which have a copy number of approximately 40. All genes are under the control of the strong T7 promoter for induction with IPTG. In the case of plasmid pAD1, the genes *ndmA* and *ndmD* are under the control of a single T7 promoter with a short synthetic ribosomal binding site between the two genes to promote translation of *ndmD*.

The *ndmA* gene was amplified by PCR from *P. putida* CBB5 genomic DNA (gDNA) with three sets of primers: ndmA-F-NcoI (5′-GCAAGGTCCATGGAGCAGGCGATCATCAATGATGA-3′) and ndmA-R-KpnI (5′-CCTCCGGGTACCTTATATGTAGCTCCTATCGCTT-3′) produced insert 1, ndmA-F-NcoI and ndmA-R-BamHI (5′-CCTCCGGGATCCTTATATGTAGCTCCTATCGCTT-3′) produced insert 2, and ndmA-F-NdeI (5′-GCACGGCATATGGAGCAGGCGATCATCAATGATGA-3′) and ndmA-R-KpnI produced insert 3. Insert 1 was cloned into the pACYCDuet-1 plasmid at the NcoI and KpnI sites, resulting in plasmid dA. This plasmid contained only one copy of *ndmA* controlled by the T7 promoter. Insert 2 was also cloned into the pACYCDuet-1 plasmid using the NcoI and BamHI sites, resulting in plasmid dA0. Plasmid dA0 contained one copy of *ndmA* and a second, empty multiple cloning site. Insert 3 was cloned into the second multiple cloning site of plasmid dA0 at the NdeI and KpnI sites, resulting in plasmid dAA.

In a similar fashion, the *ndmD* gene was also amplified from *P. putida* CBB5 gDNA by PCR using two sets of primers: ndmD-F-NcoI (5′-GTGAGATCCATGGACAAACTTGACGTCAACCAGTGG-3′) and ndmD-R-BamHI (5′-GGGACGGGGATCCTCACAGATCGAGAACGATTTTTTTGGA-3′) produced insert 4, and ndmD-F-NdeI (5′-GTGAGATCATATGAACAAACTTGACGTCAACCAGTGG-3′) and ndmD-R-KpnI (5′-GGGACGGGGTACCTCACAGATCGAGAACGATTTTTTTGGA-3′) produced insert 5. Insert 4 was cloned into the pACYCDuet-1 plasmid at the NcoI and BamHI sites, resulting in plasmid dD0, which contained one copy of *ndmD* and a second, empty multiple cloning site. Insert 5 was cloned into the empty multiple cloning site at the NdeI and KpnI sites of dD0, resulting in plasmid dDD. Insert 3 (containing *ndmA*) was also cloned into the NdeI and KpnI sites of dD0, yielding plasmid dDA. DNA sequencing of all plasmids confirmed that PCR amplification and cloning procedures did not introduce any mutations into the gene sequences.

### Strain construction

*E. coli* BL21(DE3) is the parent strain for all bacterial strains used. A list of all strains in this study is given in Table 1. Plasmids dDD, dDA, and dAA were transformed into strain pAD1, which already contained plasmid pAD1 [23], yielding strains pAD1dDD, pAD1dDA, and pAD1dAA, respectively. Plasmids dAA and dA were transformed into strain pHisD, which already contained pET28-His-ndmD [43], resulting in strains pDdAA and pDdA, respectively. Transformants were recovered on LB agar containing appropriate antibiotics at the following levels: 34 μg/mL chloramphenicol, 100 μg/mL ampicillin and 30 μg/mL kanamycin.

### Cell growth and protein expression

*E. coli* strains were grown in SB or LB medium with appropriate antibiotic at 37 °C with shaking at 250 rpm. Concentration of antibiotic used was 34, 30, and 100 µg/mL for chloramphenicol, kanamycin, and ampicillin respectively. Cell density was monitored by measuring the optical density at 600 nm (OD_600_). Upon reaching an OD_600_ of ~0.5, Ferric chloride (FeCl_3_·6H_2_O) was added (0.02 mM final concentration) and temperature was lowered to 18 °C. When the OD reached (0.8–1), IPTG was added (0.2 mM final concentration) to induce expression of *ndmA* and *ndmD*. The IPTG concentration of 0.2 mM was previously determined to give optimum protein expression [43]. Cells were harvested after (14–16) hours of induction by centrifugation at 10,000*g* for 20 min at 4 °C and washed twice in 50 mM cold potassium phosphate (KPi) buffer (pH 7.5). Pelleted cells (wet cells) were weighed and re-suspended in 50 mM KPi buffer prior to activity assays.

### Assays for 3MX and 1MX production

Other than where noted, reactions were carried out in 2 mL microcentrifuge tubes with 1 mL total reaction volume containing an initial TP concentration of 1 mM and wet cell concentration of 15 mg/mL. A VWR^®^ symphony™ Incubating Microplate Shaker was used to carry out the reaction at 30 °C and 400 rpm. 100 µL Samples were taken periodically for HPLC analysis, and concentrations of TP, 3MX and 1MX were calculated using appropriate standards. Reactions for product isolation were carried out in 1.3 L total volume with the same cell and TP concentrations as above (15 g/L and 1 mM, respectively). These large-scale reactions were carried out in an Excella E24 Incubator Shaker (Eppendorf, Hamburg, Germany) shaker at 30 °C and 250 rpm. After all TP was consumed, the post-reaction mixture was centrifuged at 10,000 x g to separate the supernatant (products) from the cells.

### Preparatory HPLC methods and product isolation

Purification of 3MX and 1MX was carried out with preparatory-scale HPLC using a Shimadzu LC-10AD HPLC system equipped with a photodiode array detector. A Hypersil BDS C18 column of 21.2 mm diameter and 25 cm length was used as the stationary phase. Methanol–water-acetic acid (5:95:0.5, vol/vol/vol) was used as the mobile phase with an optimized flow rate of 2.5 mL/min. The molecules resolved by the C18 column passed through the photodiode array detector, in which UV–visible absorption spectra were recorded. This HPLC is equipped with two pumps, A and B. The isocratic method was developed to be programmed so that pump B provided the mobile phase and pump A injected 25 mL of post-reaction mixture in 10 min periods. At the end of the preparative chromatography 900 mL 3MX solution and 700 mL 1MX solution were collected in two separate bottles. The solutions were concentrated by vacuum drying using Buchi Rotovap R114. The bath temperature was 60–70 °C. Volume reduction was 200 mL for 3MX solution and 150 mL 1MX. Both solutions were frozen to −80 °C and then were dried overnight in a Virtis Genesis 35EL freeze dryer (SP Scientific, Stone Ridge, NY) with a vacuum of 90 torr.

### Analytical procedures

Identification and quantification of TP, 3MX, and 1MX were conducted on the same HPLC system described above. A Hypersil BDS C18 column (4.6 by 125 mm) was used as the stationary phase. The same mobile phase was used with a flow rate of 0.5 mL/min. Purity of 3MX was confirmed by high resolution LC–MS facility at the University of Iowa, Department of Chemistry using a Waters Q-TOF Premier interfaced with an Acquity UPLC system. The NMR results were obtained from the NMR facility at the Chemistry Department of the University of Iowa. The spectrum was recorded in DMSO-*d*_*6*_ with a Bruker DRX 500 NMR spectrometer at 300 K. The chemical shifts were relative to DMSO-*d*_6_ using the standard δ notation in parts per million.

## Additional file

10.1186/s12934-015-0395-1 Prices of various natural methylxanthines obtained from the Sigma–Aldrich website on 18 September, 2015. **Table S2.** Estimated copy number of *ndmA* and *ndmD* genes in strains used in this study. **Table S3.** Comparison of growth and activity of resting cell suspensions of strains pDdA, pDdAA, and pAD1dDD. Concentrations of TP, 3MX, and 1MX after 90 min are reported as means with standard deviations of triplicate reactions. **Table S4.** Comparison of growth and activity of resting cell suspension of strain pDdA grown in LB and SB. Concentrations of TP, 3MX, and 1MX after 2 h are reported as means with standard deviations of triplicate reactions. **Figure S1.** Maps of plasmids in strains used to produce 3MX from TP. ori_pBR322, pBR322 origin of replication; ori_P15A, P15A origin of replication; Amp-R, ampicillin resistance gene, Kan-R, kanamycin resistance gene, CAT, chloramphenicol resistance gene, ndmA, *N*1-demethylase gene; ndmD, *N*-demethylase reductase gene; His-ndmD, N-terminal His6-tagged *N*-demethylase reductase gene; T7, T7 promoter. Plasmids beginning with “p” use pET backbones, plasmids beginning with “d” use the pACYCDuet-1 plasmid backbone. **Figure S2.** SDS-PAGE analysis of *ndmA* and *ndmD* expression in metabolically engineered strains of *E. coli*. A total of 10 μg protein was loaded into each well. Molecular weights of markers (in kDa) are shown to the left of the gel. Blue arrows indicate NdmA and NdmD protein bands. Lane 1, pAD1 soluble fraction; lane 2, pAD1 insoluble fraction; lane 3, pDdA soluble fraction; lane 4, pDdA insoluble fraction; lane 5, BL21(DE3) soluble fraction (negative control); lane 6, molecular weight standard; lane 7, pAD1DD soluble fraction; lane 8, pAD1DD insoluble fraction; lane 9, pDdAA soluble fraction; lane 10, pDdAA insoluble fraction. **Figure S3.** Separation of 3MX and 1MX by preparative chromatography. Retention times of 3MX and 1MX are 116 and 135 min, respectively. **Figure S4.** NMR of 3-methylxanthine. (A) NMR of 3MX standard obtained from Sigma Aldrich. (B) NMR of biologically produced and purified 3MX sample produced in this work

## Abbreviations

TP: theophylline
1MX: 1-methylxanthine
3MX: 3-methylxanthine
LB: Luria–Bertani broth
SB: super broth
HPLC: high pressure liquid chromatography
LC–MS: liquid chromatography–mass spectrophotometry
NMR: nuclear magnetic resonance
SDS-PAGE: sodium dodecyl sulfate–polyacrylamide gel electrophoresis

## References

[1] Ashihara H, Crozier A (1999). Biosynthesis and metabolism of caffeine and related purine alkaloids in plants. Adv Bot Res..

[2] Anaya AL, Cruz-Ortega R, Waller GR (2006). Metabolism and ecology of purine alkaloids. Front Biosci.

[3] Dash SS, Gummadi SN (2006). Catabolic pathways and biotechnological applications of microbial caffeine degradation. Biotechnol Lett.

[4] Lelo A, Miners J, Robson R, Birkett D (1986). Quantitative assessment of caffeine partial clearances in man. Br J Clin Pharmacol.

[5] Daly J (2007). Caffeine analogs: biomedical impact. Cell Mol Life Sci.

[6] Daly JW, Butts-Lamb P, Padgett W (1983). Subclasses of adenosine receptors in the central nervous system: interaction with caffeine and related methylxanthines. Cell Mol Neurobiol.

[7] Jacobson KA, Van Galen PJ, Williams M (1992). Adenosine receptors: pharmacology, structure-activity relationships, and therapeutic potential. J Med Chem.

[8] Chasin M, Harris D (1976). Inhibitory and activators of cyclic nucleotide phosphodiesterase. Adv Cyclic Nucleotide Res.

[9] Butcher R, Sutherland EW (1962). Adenosine 3′, 5′-phosphate in biological materials. I. Purification and properties of cyclic 3′, 5′-nucleotide phosphodiesterase and use of this enzyme to characterize adenosine 3′, 5′-phosphate in human urine. J Biol Chem.

[10] Bianchi C (1961). The effect of caffeine on radiocalcium movement in frog sartorius. J Gen Physiol.

[11] Frank G (1962). Utilization of bound calcium in the action of caffeine and certain multivalent cations on skeletal muscle. J Physiol.

[12] Daly JW (2000). Alkylxanthines as research tools. J Auton Nerv Syst.

[13] Shi D, Padgett WL, Daly JW (2003). Caffeine analogs: effects on ryanodine-sensitive calcium-release channels and GABAA receptors. Cell Mol Neurobiol.

[14] Marangos P, Paul SM, Parma A, Goodwin F, Syapin P, Skolnick P (1979). Purinergic inhibition of diazepam binding to rat brain (in vitro). Life Sci.

[15] Williams JF, Lowitt S, Polson JB, Szentivanyi A (1978). Pharmacological and biochemical activities of some monomethylxanthine and methyluric acid derivatives of theophylline and caffeine. Biochem Pharmacol.

[16] Birkett D, Dahlqvist R, Miners J, Lelo A, Billing B (1985). Comparison of theophylline and theobromine metabolism in man. Drug Metab Disposition.

[17] Lee SW, Zhao L, Pardi A, Xia T (2010). Ultrafast dynamics show that the theophylline and 3-methylxanthine aptamers employ a conformational capture mechanism for binding their ligands. Biochemistry.

[18] Desai SK, Gallivan JP (2004). Genetic screens and selections for small molecules based on a synthetic riboswitch that activates protein translation. J Am Chem Soc.

[19] Tang-Liu D, Williams R, Riegelman S (1983). Disposition of caffeine and its metabolites in man. J Pharmacol Exp Ther.

[20] Scott N, Chakraborty J, Marks V (1986). Urinary metabolites of caffeine in pregnant women. Br J Clin Pharmacol.

[21] Birkett D, Miners J, Day R (1991). 1-Methylxanthine derived from theophylline as an in vivo biochemical probe of allopurinol effect. Br J Clin Pharmacol.

[22] Birkett DJ, Miners JO, Valente L, Lillywhite K, Day R (1997). 1-Methylxanthine derived from caffeine as a pharmacodynamic probe of oxypurinol effect. Br J Clin Pharmacol.

[23] Summers RM, Mohanty SK, Gopishetty S, Subramanian M (2015). Genetic characterization of caffeine degradation by bacteria and its potential applications. Microb Biotechnol.

[24] Taylor EC, Barton JW, Paudler WW (1961). Studies in purine chemistry. X. Some derivatives of 9-aminopurines. J Org Chem.

[25] Shamim MT, Ukena D, Padgett WL, Daly JW (1989). Effects of 8-phenyl and 8-cycloalkyl substituents on the activity of mono-, di, and trisubstituted alkylxanthines with substitution at the 1-, 3-, and 7-positions. J Med Chem.

[26] Gopishetty S, Louie T, Yu C, Subramanian M. Microbial degradation of caffeine, methylxanthines, and its biotechnological applications. In: Thatoi H, Mishra B, editors. Microbial biotechnology methods and applications. New Delhi: Narosa Publishing Houst Pvt, Ltd; 2011. p. 44–67.

[27] Traube W (1900). Der synthetische aufbau der harnsäure, des xanthins, theobromins, theophyllins und caffeïns aus der cyanessigsäure. Ber Dtsch Chem Ges.

[28] Yoneda F, Higuchi M, Mori K, Senga K, Kanamori Y, Shimizu K, Nishigaki S (1978). Synthesis of xanthines by dehydrogenative cyclization of 6-amino-5-benzylideneaminouracils with diethyl azodicarboxylate. Chem Pharm Bull (Tokyo).

[29] Lister JH. The chemistry of heterocyclic compounds, The Purines: Supplement 1. Wiley-Interscience; 2009.

[30] Zavialov IA, Dahanukar VH, Nguyen H, Orr C, Andrews DR (2004). New and Practical Method for Synthesis of 1-and 1, 3-Substituted Xanthines. Org Lett.

[31] Allwood MB, Cannan B, van Aalten DMF, Eggleston IM (2007). Efficient synthesis of 1, 3, 7-substituted xanthines by a safety-catch protection strategy. Tetrahedron.

[32] Liu G, Reddy PSMM, Barber JR, Ng SC, Zhou Y (2010). Synthesis of Novel 3, 7-Dihydro-purine-2, 6-dione Derivatives. Synth Commun.

[33] Hollingsworth RG, Armstrong JW, Campbell E (2002). Pest control: caffeine as a repellent for slugs and snails. Nature.

[34] Nathanson JA (1984). Caffeine and related methylxanthines: possible naturally occurring pesticides. Science.

[35] Dash SS, Gummadi SN (2010). Biodegradation of caffeine by Pseudomonas sp. NCIM 5235. Res J Microbiol.

[36] Gokulakrishnan S, Chandraraj K, Gummadi SN (2007). A preliminary study of caffeine degradation by Pseudomonas sp. GSC 1182. Int J Food Microbiol.

[37] Yu CL, Kale Y, Gopishetty S, Louie TM, Subramanian M (2008). A novel caffeine dehydrogenase in *Pseudomonas* sp. strain CBB1 oxidizes caffeine to trimethyluric acid. J Bacteriol.

[38] Yu CL, Louie TM, Summers R, Kale Y, Gopishetty S, Subramanian M (2009). Two distinct pathways for metabolism of theophylline and caffeine are coexpressed in *Pseudomonas putida* CBB5. J Bacteriol.

[39] Yu CL, Summers RM, Li Y, Mohanty SK, Subramanian M, Pope RM (2014). Rapid identification and quantitative validation of a caffeine-degrading pathway in *Pseudomonas* sp. CES. J Proteome Res.

[40] Mazzafera P, Olsson O, Sandberg G (1996). Degradation of caffeine and related methylxanthines bySerratia marcescens isolated from soil under coffee cultivation. Microb Ecol.

[41] Woolfolk C (1975). Metabolism of N-methylpurines by a *Pseudomonas putida* strain isolated by enrichment on caffeine as the sole source of carbon and nitrogen. J Bacteriol.

[42] Madyastha K, Sridhar G (1998). A novel pathway for the metabolism of caffeine by a mixed culture consortium. Biochem Biophys Res Commun.

[43] Summers RM, Louie TM, Yu C-L, Gakhar L, Louie KC, Subramanian M (2012). Novel, highly specific N-demethylases enable bacteria to live on caffeine and related purine alkaloids. J Bacteriol.

[44] Summers R, Gopishetty S, Mohanty S, Subramanian M (2014). New genetic insights to consider coffee waste as feedstock for fuel, feed, and chemicals. Cent Eur J Chem.

[45] BuyersGuideChem. http://www.buyersguidechem.com/AliefAus.php?pnumm=530921731638. Accessed 28 Oct 2015.

[46] Summers RM, Seffernick JL, Quandt EM, Yu CL, Barrick JE, Subramanian MV (2013). Caffeine junkie: an unprecedented glutathione S-transferase-dependent oxygenase required for caffeine degradation by *Pseudomonas putida* CBB5. J Bacteriol.

[47] Lee Y-L, Su M-S, Huang T-H, Shaw J-F (1999). C-terminal His-tagging results in substrate specificity changes of the thioesterase I from *Escherichia coli*. J Am Oil Chem Soc.

[48] Hakil M, Denis S, Viniegra-Gonzalez G, Augur C (1998). Degradation and product analysis of caffeine and related dimethylxanthines by filamentous fungi. Enzyme Microb Technol.

[49] Asano Y, Komeda T, Yamada H (1993). Microbial production of theobromine from caffeine. Biosci Biotechnol Biochem.

[50] Dash SS, Gummadi SN (2007). Degradation kinetics of caffeine and related methylxanthines by induced cells of *Pseudomonas* sp. Curr Microbiol.

[51] Jin L, Bhuiya MW, Li M, Liu X, Han J, Deng W, Wang M, Yu O, Zhang Z (2014). Metabolic engineering of *Saccharomyces cerevisiae* for caffeine and theobromine production. PLoS One.

[52] Udayasankar K, Manohar B, Chakkalingam A (1986). A note on supercritical carbon dioxide decaffeination of coffee. J Food Sci Technol.

[53] Ramalakshmi K, Raghavan B (1999). Caffeine in coffee: its removal. Why and how?. Crit Rev Food Sci Nutr.

[54] Bobranski B, Synowiedski Z (1948). New syntheses of caffeine and theophylline. J Am Pharm Assoc.

[55] Hu X, Wan X, Bal R, Yang H (2003). Theobromine and caffeine recovery with solvent extraction. Sep Sci Technol.

[56] Saldaña MD, Mohamed RS, Baer MG, Mazzafera P (1999). Extraction of purine alkaloids from mate (Ilex paraguariensis) using supercritical CO_2_. J Agric Food Chem.

[57] Human metabolome database 3-methylxanthine spectrum. http://www.hmdb.ca/spectra/ms_ms/5549. Accessed 12 Nov 2015.

