# Supplementary material for: Direct conversion of theophylline to 3-methylxanthine by metabolically engineered E. coli
Source: Microb Cell Fact. 2015 Dec 21;14:203. doi: 10.1186/s12934-015-0395-1 (PMC4687300; doi:10.1186/s12934-015-0395-1)
Supplement: Supplementary file 1 — 10.1186/s12934-015-0395-1 Prices of various natural methylxanthines obtained from the Sigma–Aldrich website on 18 September, 2015. Table S2. Estimated copy number of ndmA and ndmD genes in strains used in this study. Table S3. Comparison of growth and activity of resting cell suspensions of strains pDdA, pDdAA, and pAD1dDD. Concentrations of TP, 3MX, and 1MX after 90 min are reported as means with standard deviations of triplicate reactions. Table S4. Comparison of growth and activity of resting cell suspension of strain pDdA grown in LB and SB. Concentrations of TP, 3MX, and 1MX after 2 h are reported as means with standard deviations of triplicate reactions. Figure S1. Maps of plasmids in strains used to produce 3MX from TP. ori_pBR322, pBR322 origin of replication; ori_P15A, P15A origin of replication; Amp-R, ampicillin resistance gene, Kan-R, kanamycin resistance gene, CAT, chloramphenicol resistance gene, ndmA, N1-demethylase gene; ndmD, N-demethylase reductase gene; His-ndmD, N-terminal His6-tagged N-demethylase reductase gene; T7, T7 promoter. Plasmids beginning with “p” use pET backbones, plasmids beginning with “d” use the pACYCDuet-1 plasmid backbone. Figure S2. SDS-PAGE analysis of ndmA and ndmD expression in metabolically engineered strains of E. coli. A total of 10 μg protein was loaded into each well. Molecular weights of markers (in kDa) are shown to the left of the gel. Blue arrows indicate NdmA and NdmD protein bands. Lane 1, pAD1 soluble fraction; lane 2, pAD1 insoluble fraction; lane 3, pDdA soluble fraction; lane 4, pDdA insoluble fraction; lane 5, BL21(DE3) soluble fraction (negative control); lane 6, molecular weight standard; lane 7, pAD1DD soluble fraction; lane 8, pAD1DD insoluble fraction; lane 9, pDdAA soluble fraction; lane 10, pDdAA insoluble fraction. Figure S3. Separation of 3MX and 1MX by preparative chromatography. Retention times of 3MX and 1MX are 116 and 135 min, respectively. Figure S4. NMR of 3-methylxanthine. (A) NMR of 3MX sta [file 12934_2015_395_MOESM1_ESM.pdf]

Supplementary Information for

Direct Conversion of Theophylline to 3-Methylxanthine by Metabolically Engineered *E. coli*

Khalid H. R. Al-Gharrawi[1,4], Ryan M. Summers[2], Sridhar Gopishetty[3], and Mani Subramanian[1*]

[1]Department of Chemical and Biochemical Engineering, The University of Iowa, Coralville, IA 52241, USA.

[2]Department of Chemical and Biological Engineering, The University of Alabama, Tuscaloosa, AL, 35487, USA.

[3]Center for Biocatalysis and Bioprocessing, The University of Iowa, Coralville, IA 52241, USA.

[4]Department of Chemical Engineering, University of Baghdad, Baghdad, Iraq

*For correspondence. E-mail mani-subramanian@uiowa.edu; Tel.858-342-6904; Fax 319-335-4901.

Methylxanthine Sources

Prices of various natural methylxanthines were obtained from the Sigma-Aldrich website on 18 September, 2015, and are listed in Table S1. Caffeine is produced principally by extraction from plant matter (i.e., coffee decaffeination), and is available in bulk quantities (up to 25 kg). The remaining compounds are primarily produced through chemical synthesis. The maximum quantity of 1-, 3-, and 7-methylxanthines is 1 g, while the maximum available quantity of paraxanthine is only 0.5 g. For uniformity, retail price of the largest available quantity is listed.

Table S1. Prices of various natural methylxanthines obtained from the Sigma-Aldrich website on 18 September, 2015.

Compound	Catalog Number	Quantity (g)	Price (US$)	Price per unit mass (US$/g)
Caffeine	W222402-25KG-K	25000	$ 1,049.00	$ 0.04
Theophylline	T1633-1KG	1000	$ 186.00	$ 0.19
Theobromine	T4500-100G	100	$ 71.00	$ 0.71
Paraxanthine	D5385-500MG	0.5	$ 577.00	$ 1,154.00
1-Methylxanthine	69720-1G	1	$ 845.00	$ 845.00
3-Methylxanthine	222526-1G	1	$ 234.00	$ 234.00
7-Methylxanthine	69723-1G	1	$ 466.50	$ 466.50

Table S2. Estimated copy number of *ndmA* and *ndmD* genes in strains used in this study.

	Approximate gene copy number*		
Strain	*ndmA*	*ndmD*	*ndmD*:*ndmA* ratio
pAD1	40	40	1.0
pAD1dAA	60	40	0.67
pAD1dDA	50	50	1.0
pAD1dDD	40	60	1.5
dDA	10	10	1.0
pDdAA	20	40	2.0
pDdA	10	40	4.0

* Approximate gene copy number was estimated based on approximate copy number of the plasmid backbone (40 for pAD1 and pET28-His-ndmD, 10 for dA, dAA, dDD, and dDA) and number of genes in each plasmid. This value was calculated as $C_i = \sum N_{ij} P_{ij}$, where C_i = gene copy number, N_{ij} = number of genes i on plasmid j, P_j = copy number of plasmid j backbone, i = gene (*ndmA* or *ndmD*), and j = plasmid backbone (pET or pACYCDuet-1). Plasmid copy numbers used are based on values reported by the manufacturer [1] and have not been determined experimentally in this study.

Resting Cell Assays

Cells were harvested by centrifugation, washed twice in 50 mM potassium phosphate (KPi) buffer (pH 7.5) and re-suspended in KPi buffer prior to activity assays. Resting cell assays were performed at 30°C with 400 rpm shaking in a VWR® symphony™ Incubating Microplate Shaker. Reactions were carried out in 2 mL microcentrifuge tubes with 1 mL total volume. Initial activity screening for strains pDdA, pDdAA, and pAD1dDD (Table S3) was carried out with 30 mg/mL cells and an initial TP concentration of 4 mM. Further screening of activity from strain pDdA grown in different media was carried out with 15 mg/mL cells and an initial TP concentration of 1 mM (Table S4).

Table S3. Comparison of growth and activity of resting cell suspensions of strains pDdA, pDdAA, and pAD1dDD. Concentrations of TP, 3MX, and 1MX after 90 min are reported as means with standard deviations of triplicate reactions.

Strain	Final OD_{600}	Final cell mass (g)	Final [TP] (mM)	Final [3MX] (mM)	Final [1MX] (mM)
pDdA	6.2	0.75	1.52 ± 0.27	2.25 ± 0.31	0.42 ± 0.05
pDdAA	6.3	0.84	1.70 ± 0.83	1.84 ± 0.74	0.40 ± 0.15
pAD1dDD	6.7	0.81	2.03 ± 0.54	1.59 ± 0.55	0.37 ± 0.10

Table S4. Comparison of growth and activity of resting cell suspension of strain pDdA grown in LB and SB. Concentrations of TP, 3MX, and 1MX after 2 h are reported as means with standard deviations of triplicate reactions.

Media	Final OD_{600}	Final cell mass (g)	Final [TP] (mM)	Final [3MX] (mM)	Final [1MX] (mM)
LB	4.75	0.6	0.02 ± 0.02	0.83 ± 0.02	0.12 ± 0.00
SB	7.5	0.9	0.00 ± 0.00	0.82 ± 0.00	0.13 ± 0.00

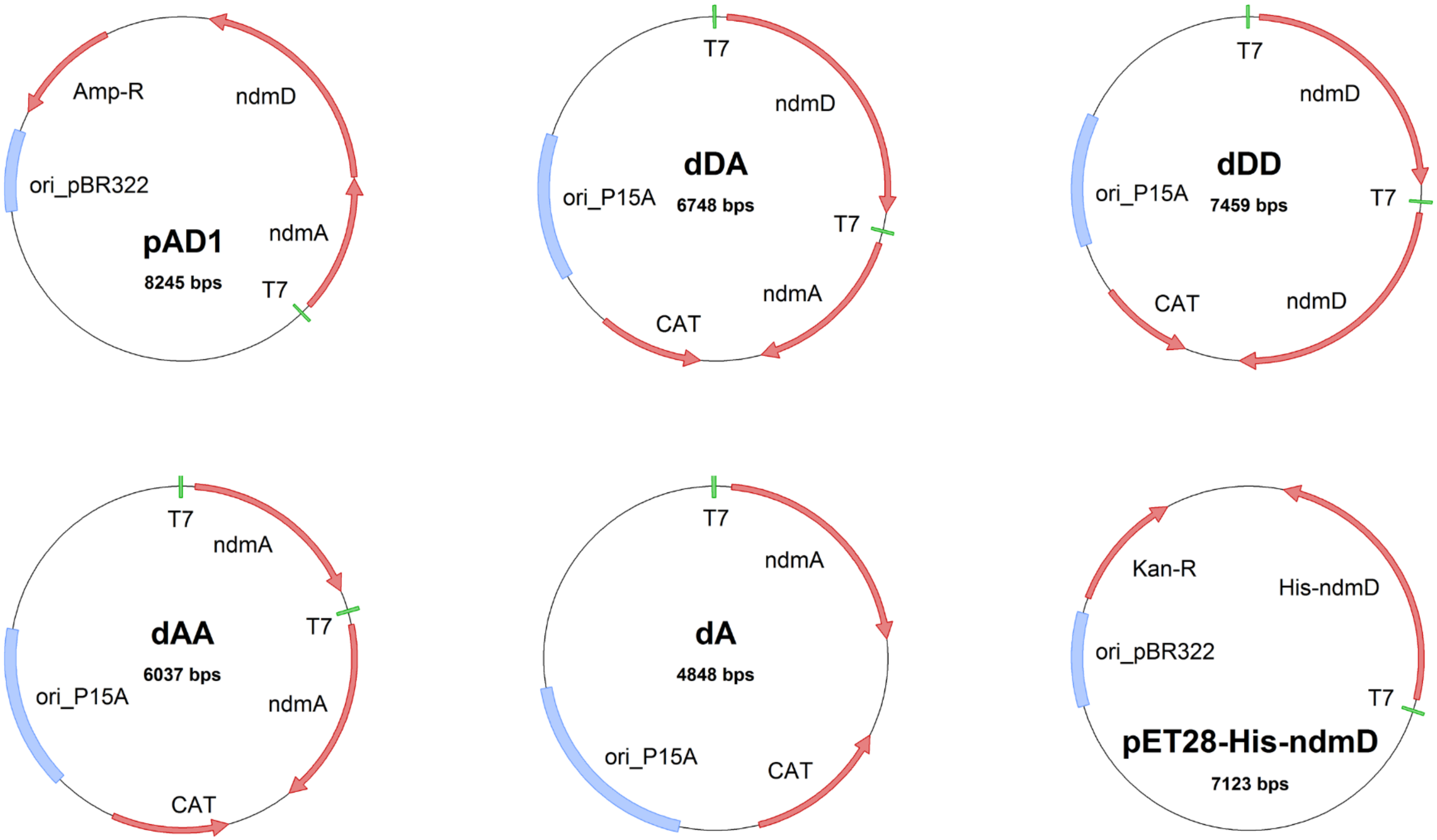

Figure S1. Maps of plasmids in strains used to produce 3MX from TP. ori_pBR322, pBR322 origin of replication; ori_P15A, P15A origin of replication; Amp-R, ampicillin resistance gene, Kan-R, kanamycin resistance gene, CAT, chloramphenicol resistance gene, ndmA, N_1-demethylase gene; ndmD, *N*-demethylase reductase gene; His-ndmD, N-terminal His_6-tagged *N*-demethylase reductase gene; T7, T7 promoter. Plasmids beginning with “p” use pET backbones, plasmids beginning with “d” use the pACYCDuet-1 plasmid backbone.

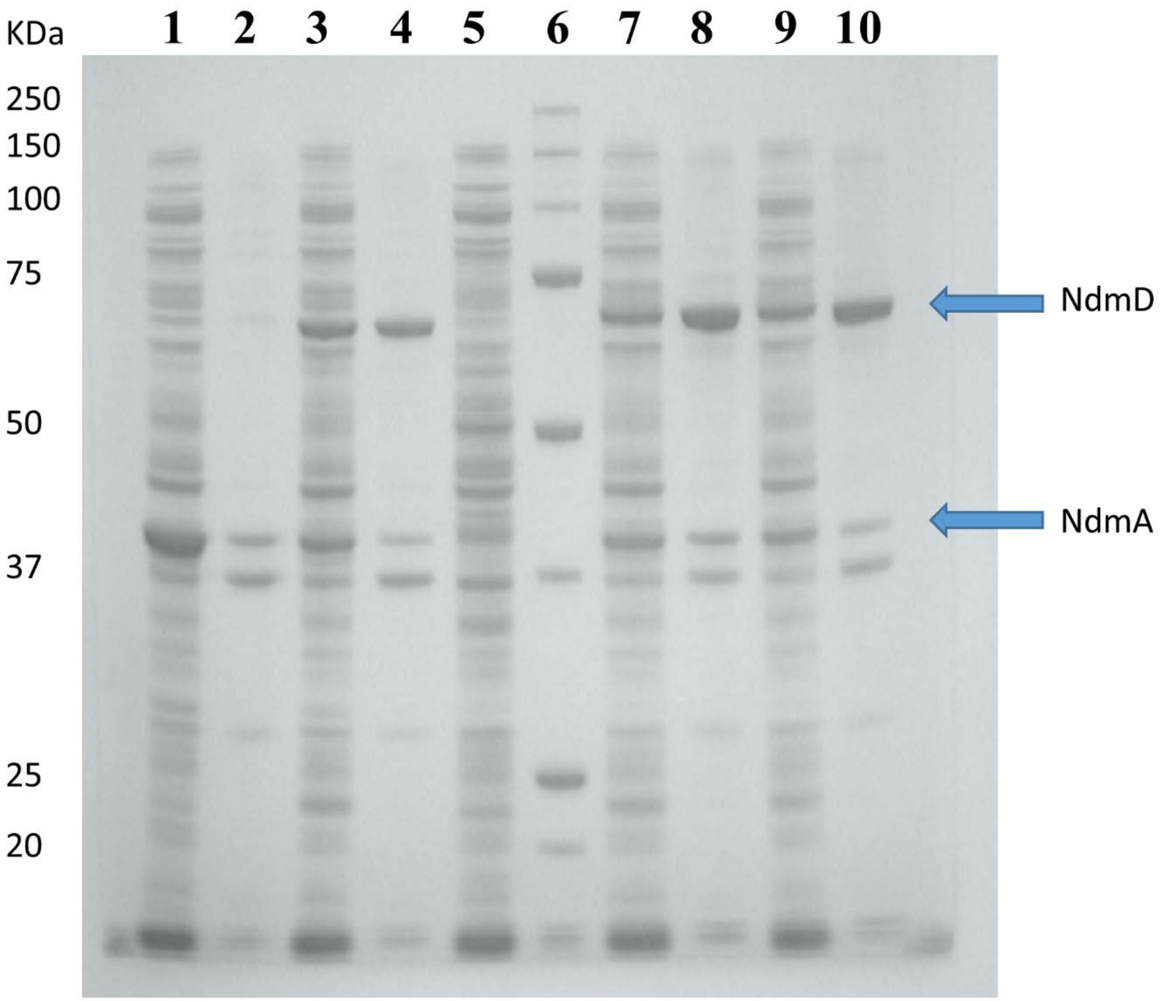

Figure S2. SDS-PAGE analysis of *ndmA* and *ndmD* expression in metabolically engineered strains of *E. coli*. A total of 10 µg protein was loaded into each well. Molecular weights of markers (in kDa) are shown to the left of the gel. Blue arrows indicate NdmA and NdmD protein bands. Lane 1, pAD1 soluble fraction; lane 2, pAD1 insoluble fraction; lane 3, pDdA soluble fraction; lane 4, pDdA insoluble fraction; lane 5, BL21(DE3) soluble fraction (negative control); lane 6, molecular weight standard; lane 7, pAD1DD soluble fraction; lane 8, pAD1DD insoluble fraction; lane 9, pDdAA soluble fraction; lane 10, pDdAA insoluble fraction.

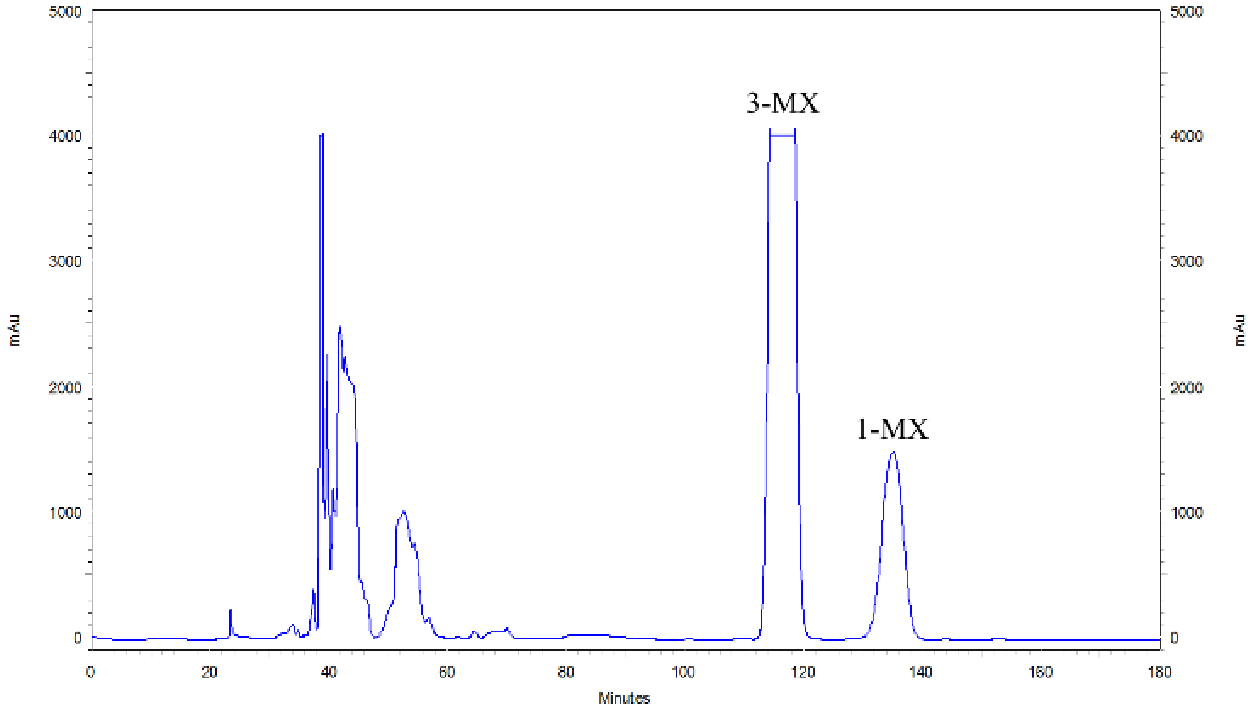

Figure S3. Separation of 3MX and 1MX by preparative chromatography. Retention times of 3MX and 1MX are 116 and 135 min, respectively.

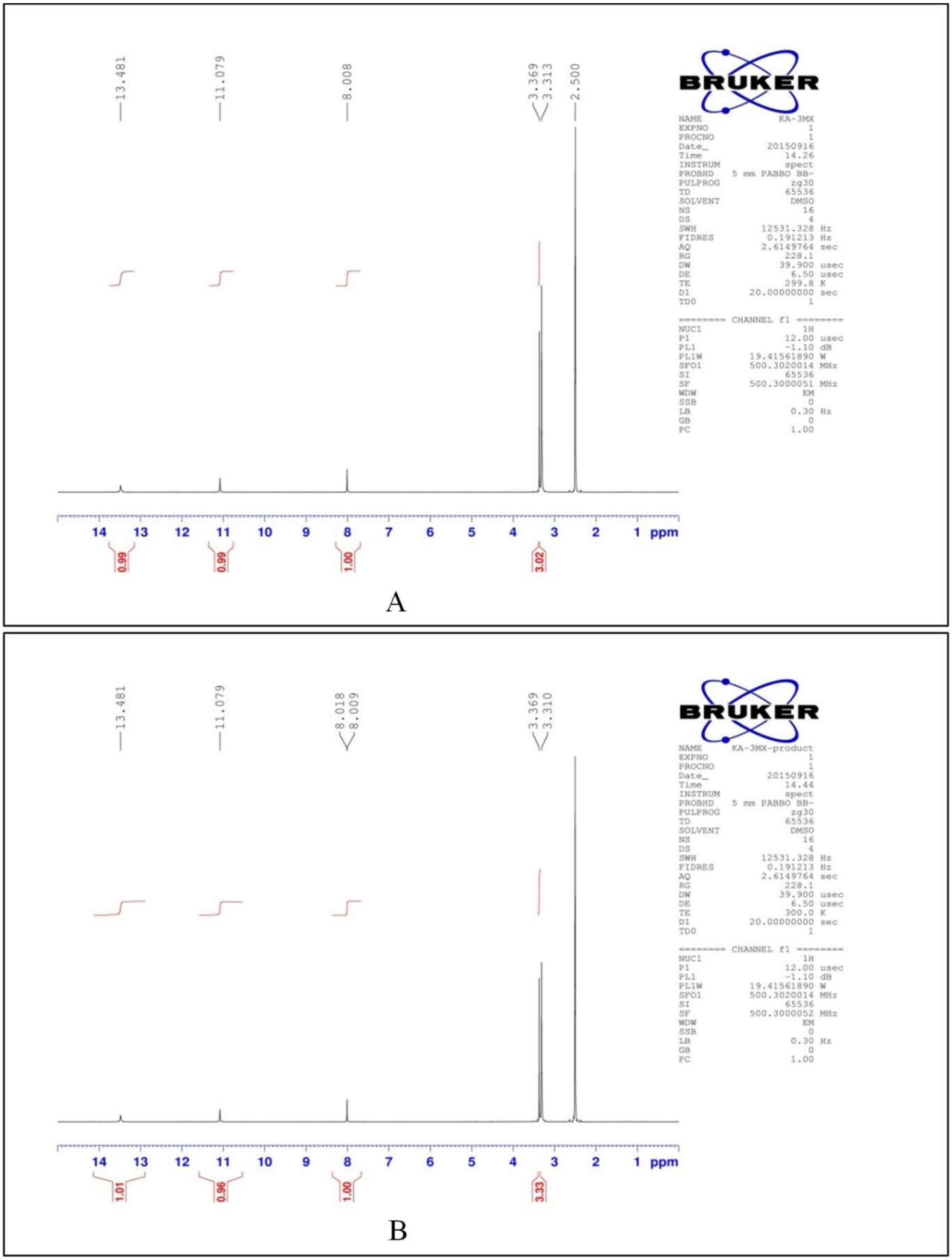

Figure S4. NMR of 3-methylxanthine. (A) NMR of 3MX standard obtained from Sigma Aldrich. (B) NMR of biologically produced and purified 3MX sample produced in this work.

Supplemental References

1. Held D, Yaeger K, Novy R: **New coexpression vectors for expanded compatibilities in E. coli.** *Innovations* 2003, **18:**4-6.
